# Neural personal information and its legal protection: evidence from China

**DOI:** 10.1093/jlb/lsaf006

**Published:** 2025-04-15

**Authors:** Bin Wei, Shuyao Cheng, Yang Feng

**Affiliations:** Guanghua School of Law, Law & Al Lab, Zhejiang University, No. 51 Zhijiang Road, Hangzhou, Zhejiang 310008, P.R. China; Guanghua School of Law, Zhejiang University, No. 51 Zhijiang Road, Hangzhou, Zhejiang, P.R. China; Guanghua School of Law, Zhejiang University, No. 51 Zhijiang Road, Hangzhou, Zhejiang, P.R. China

**Keywords:** neural personal information, legal protection, neurotechnology, brain–computer interface

## Abstract

The rapid advancements in neuroscience highlight the pressing need to safeguard neural personal information (NPI). China has achieved significant progress in brain–computer interface technology and its clinical applications. Considering the intrinsic vulnerability of NPI and the paucity of legal scrutiny concerning NPI breaches, a thorough assessment of the adequacy of China’s personal information protection legislation is essential. This analysis contends that NPI should be classified as sensitive personal information. The absence of bespoke provisions for NPI in current legislation underscores persistent challenges in its protection. To address these gaps, this work proposes the establishment of a concentric-circle hard–soft law continuum to support a hybrid governance model for NPI, rooted in fundamental human rights principles.

## I. INTRODUCTION

Neurotechnology can offer insights into brain activity and even influence nervous system function. Brain–computer interfaces (BCIs), a key example of neurotechnology, have achieved remarkable progress in medicine, with their applications now extending beyond clinical settings.^1^ Given the promising future of BCIs, several strategic BCI plans have been launched, such as the U.S. BRAIN Initiative, the E.U. Human Brain Project, and the China Brain Project. Clinical trials for BCIs are progressing steadily. In August 2024, Neuralink successfully implanted a BCI in a second patient, with the implant demonstrating effective functioning.^2^ Nonetheless, BCIs may pose significant risks to neural personal information (NPI). NPI encompasses diverse brain data obtained through neurotechnologies, such as BCI data, electroencephalography (EEG) information, and functional magnetic resonance imaging (fMRI) data. This data can reveal highly sensitive personal information, including individuals’ thoughts, emotional states, decision-making processes, and even their preferences or intentions.^3^ The complexity of BCI technology may prevent users from fully understanding what information is being extracted from their brains and how it is subsequently utilized, creating opportunities for misuse of their NPI by data processors.^4^

Meanwhile, BCI technology has introduced the possibility of ‘hacking’ into the brain., In the context of neurotechnology, vast amounts of NPI could be harvested through data trials.^5^ Currently, BCI devices lack adequate security measures to effectively safeguard users’ NPI.^6^ Malicious BCI applications could allow hackers to monitor users’ brain activities and access NPI without their consent.^7^ Researchers have confirmed that during data processing, hackers may use machine learning algorithms to acquire substantial amounts of NPI and infer private details about users through analysis.^8^ Nevertheless, specific legal or technical measures to protect NPI from data mining and privacy invasions are still lacking.^9^

Protecting privacy rights in the era of neurotechnology has become a global legal challenge. Research has analyzed existing legal protections for mental data, focusing specifically on the E.U. General Data Protection Regulation (GDPR).^10^ Findings corroborate that while brain data may fall under GDPR provisions, these regulations may not sufficiently shield data subjects from broader BCI-related privacy issues.^11^ Current research on BCI privacy protection encompasses secure data storage and transmission,^12^ informed consent procedures,^13^ data privacy concerns,^14^ and the establishment of ethical and legal frameworks.^15^ Nonetheless, most of this research remains theoretical and technologically oriented, lacking concrete legal strategies to safeguard NPI in practical applications.

China is a leading nation in BCI research and development, bolstered by substantial government support, numerous innovative outcomes, and extensive application scenarios. During the 13th Five-Year Plan, the ‘China Brain Project’ was initiated to advance brain science and brain-like intelligence technology.^16^ Subsequently, the 14th Five-Year Plan introduced the ‘One Body, Two Wings’ initiative, which focuses on brain-inspired computing and brain–computer integration. Under national policies, Chinese research teams have achieved notable progress in various domains, including neuron-like electronics,^17^ novel electrodes for BCIs,^18^ and BCIs based on steady-state visually evoked potentials.^19^ These advancements have paved the way for BCI applications in diverse areas such as stroke rehabilitation,^20^ mental health interventions,^21^ and sleep quality enhancement.^22^ This highlights the growing significance of BCIs as therapeutic tools for addressing an range of health-related challenges.

Nonetheless, using BCIs in China presents potential risks of NPI leakage, necessitating appropriate information protection regulations. China has recently introduced various laws and regulations across criminal, civil, and administrative domains, with a growing focus on information protection and privacy legislation. The personal information protection legislation of the country can potentially provide guidance for safeguarding NPI in the context of BCIs. Nevertheless, existing Chinese laws offer few direct and explicit provisions for NPI, which may limit their practical effectiveness in ensuring robust protection.

Considering the current research gaps, the present research seeks to thoroughly examine the applicability of the personal information protection legislation of China to NPI. Additionally, this work reviews the current state of BCI technology in the country, analyzes the implementation and deficiencies of the nation’s personal information protection legislation, and contemplates strategies for protecting NPI from infringement and abuse in BCI applications. According to these findings, this study presents several targeted recommendations to establish a more robust and comprehensive regulatory framework for NPI protection in China.

This paper is structured as follows. Section II summarizes recent advancements in BCI technology in China and describes the state of commercialized BCI applications. Section III tackles the personal information protection legislation of the country, examining specific provisions and real-life examples from criminal, civil, and administrative law. Section IV assesses the suitability of the nation’s personal information protection framework for NPI, focusing on its core functions and identifying deficiencies in areas such as informed consent, information processing, and public authority oversight. Section V proposes strategies to bolster NPI protection by establishing a concentric-circle hard–soft law continuum, fostering mixed governance of NPI. Finally, Section VI concludes with a summary of the key points discussed throughout the paper.

## II. BCI TECHNOLOGY DEVELOPMENT IN CHINA

Over the past decade, China has implemented a series of supportive policies to advance BCI technology. In 2011, the Chinese Ministry of Science and Technology launched the ‘National Twelfth Five-Year Plan for Science and Technology Development,’ designating brain science as a key research priority in basic sciences.^23^ In 2021, the ministry officially initiated the ‘Brain Science and Brain-like Research’ project under the Science and Technology Innovation 2030 major initiative, which covers 59 research fields and directions with a projected national investment exceeding 3.148 billion RMB.^24^ In 2023, the establishment of the China BCI Industry Alliance marked a significant milestone in fostering interdisciplinary collaboration within the BCI field. Driven by these supportive policies, China’s BCI industry has experienced substantial growth. Over the past decade, the issuance of BCI-related patents in the country has consistently increased, with the average annual output exceeding 100 patents in the last three years alone.^25^ As of the first quarter of 2023, China is home to over 500 globally recognized BCI companies, with more than 20% of them based in the country, placing China at the forefront of the global BCI industry.^26^ By August 20, 2023, total financing in the nation’s primary BCI market surpassed 5 billion RMB, attracting investment from more than 150 institutions.^27^ Currently, the BCI market in China is valued at ~1 billion RMB.^28^

BCIs, based on the method of electroencephalogram (EEG) signal acquisition, are classified into three types: invasive, non-invasive, and semi-invasive. Chinese researchers have made notable strides across each category.

In invasive BCIs, where electrodes are implanted directly into the brain to capture neural signals, China has achieved substantial advancements. In this field, Chinese scholars have engineered flexible micro-array electrodes that snugly fit the contoured surfaces of the brain to optimize brain function protection.^29^ In early 2020, Zhejiang researchers performed China’s first invasive BCI surgery, enabling patients to precisely control external devices via brain signals.^30^ In 2023, the world’s first non-human primate interventional BCI test was successfully conducted in Beijing, signifying a major breakthrough in the country’s invasive BCI technology.^31^

Conversely, non-invasive BCIs do not employ brain implants but rather capture and decode brain signals through wearable devices attached to the scalp. In this area, China has developed a deep learning method specifically for motor imagery BCIs,^32^ created advanced neural network models,^33^ and achieved promising results in EEG-based emotion recognition^34^ and identity verification.^35^ The country’s non-invasive BCIs are applied in areas such as supporting functional recovery,^36^ controlling external devices,^37^ and monitoring user status.^38^ Notably, the China Rehabilitation Research Center, in partnership with BrainCo, has created a wearable brainwave rehabilitation system for children with autism, demonstrating positive results.^39^

Semi-invasive BCIs, which only implant electrodes on the surface of the scalp, employ cortical electroencephalography (ECoG) technology for signal analysis. A portable wireless EEG system utilizing ECoG technology has been constructed, featuring a 32-channel flexible electrode array implant that considerably enhances signal accuracy.^40^ At the World Artificial Intelligence Conference 2021, NeuroXess unveiled China’s first medical-grade semi-invasive BCI device, which has subsequently undergone validation for human clinical applications.

With these technological advancements, the commercial application of BCIs is steadily increasing. From 2019 to 2022, the global BCI market expanded from 1.2 billion USD to 1.74 billion USD, with projections affirming that it could reach 3.3 billion USD by 2027.^41^ Presently, the primary application of BCI technology in China lies within the medical and healthcare sector, constituting ~47.62% of the market, followed by consumer electronics, education, entertainment, and other sectors, with market shares of 28.57%, 13.10%, 4.76%, and 5.95%, respectively.^42^

BCI technology offers a groundbreaking approach to modulate interactions between the nervous system and its internal and external environments, providing an alternative to traditional surgical, pharmacological, and physical therapies. BCIs play a crucial role in restoring brain–limb connections, allowing neural recovery of motor functions, which are difficult to attain through traditional methods.^43^ Furthermore, BCIs can record neural activity patterns in real time, facilitating timely diagnosis and personalized treatment of neurological disorders.^44^ Additionally, BCIs have the potential for emotion identification, helping in the analysis of mood fluctuations in depression patients, thus providing valuable insights for interactions between doctors and patients.^45^ In the next phase, BCIs are anticipated to enable the integration of knowledge, emotion, and memory, advancing from human–computer symbiosis to human–computer twinning.

Generally, the comprehensive support of the Chinese government for BCI technology has considerably propelled its rapid advancement and widespread application across China. Nonetheless, as BCI technology evolves, safety and ethical concerns are increasingly evident. Ensuring the reliability and compatibility of BCI technology and addressing privacy issues related to the collection of NPI are essential for its sustainable development. As BCI technology becomes increasingly prevalent, focused attention and effective solutions for these challenges are imperative.

## III. CHINA’S PERSONAL INFORMATION PROTECTION LEGISLATION

The legislative framework of China for personal information protection began developing in the early 21st century in response to the rapid growth of Internet technology and the rising misuse and leakage of information. This development has unfolded in four key phases: (1) Starting in 2009, exploratory legislation on personal information protection within the Criminal Law; (2) From 2012 to 2021, multi-level legislation introduced laws like the *Provisions on Protecting the Personal Information of Telecommunications and Internet Users*, the *Cybersecurity Law*, and the *Data Security Law*; (3) In January 2021, the Civil Code introduced uniform provisions on personal information protection in the private law domain; (4) In August 2021, the *Personal Information Protection Law* (PIPL) was enacted, seeking to integrate regulatory frameworks for personal information across public and private law domains. Since then, China has established a comprehensive multilayered personal information protection system centered around the PIPL, the Civil Code, and relevant provisions of criminal law.

### III.A. Criminal Protection of Personal Information

In China, criminal law stands out as one of the earliest and most robust legal tools for protecting personal information, playing a central role in combating information theft.^46^ In 2009, facing a surge in information crimes spurred by rapid Internet growth, Amendment (VII) to the Criminal Law introduced penalties for illegally acquiring, providing, or selling citizens’ personal information. This is the first time Chinese legislation has set a legal limit for the processing of personal information through prohibitions. Additionally, in 2015, Amendment (IX) to the Criminal Law integrated these provisions, establishing the crime of infringing on civil personal information. In 2017, the Interpretation of the Supreme People’s Court and the Supreme People’s Procuratorate on Several Issues concerning the Application of Law in the Handling of Criminal Cases of Infringing on Citizens’ Personal Information was issued, considering the type and sensitivity of personal information as factors in conviction and sentencing. Together, these measures establish a dual-layered model of personal information protection within China’s criminal law, reinforced by judicial interpretations.^47^

In protecting personal information, the country’s criminal law highlights combating the illegal acquisition of information with a value orientation prioritizing social order, followed by industrial development interests, and individual rights thereafter.^48^ The legal interests protected by criminal law encompass personal information rights and interests and the state’s order for the management of personal information security, with the latter being the primary legal interest.^49^ A quantitative review of diverse criminal judgments in criminal cases encompassing the infringement of citizens’ personal information affirms that courts are more likely to be lenient in sentencing and lack precision in identifying the sensitivity levels of personal information.^50^ For instance, an analysis of 507 judgments in identity theft criminal cases in Shanghai courts highlights that judges commonly possess considerable discretion to deviate from the sentencing standards stipulated by the Criminal Code and the 2017 Judicial Interpretation.^51^

NPI is identifiable and may contain sensitive information. Nonetheless, in criminal justice, NPI is generally treated as physiological health data and receives only standard protection.^52^ Acts that infringe upon NPI are commonly covert, making them difficult to detect. Even when the circumstances of these acts are serious, classifying them as crimes is challenging because the Chinese criminal law has not set standards for their conviction. Even if such offenses are eventually recognized as crimes, the absence of specific legal guidelines or case law for sentencing creates ambiguity. This lack of clarity may lead to confusion in the trial process, potentially compromising fairness and efficiency.

### III.B. Civil Protection of Personal Information

China’s civil law provisions on personal information protection began later than its criminal law efforts but are more comprehensive. In 2013, the Law of the People’s Republic of China on the Protection of Consumer Rights and Interests was enacted, introducing new special provisions specifically focused on consumer information protection and marking the commencement of regulating personal information. The *General Provisions of the Civil Law of the People’s Republic of China*, established in 2017, was the first to recognize personal information as part of civil protection. Later, in 2021, the implementation of the *Civil Code* provided a crucial legal foundation for citizens to assert their rights to personal information. The *Civil Code* integrates a synergistic model of ‘information rights and interests’ with ‘privacy rights.’ In situations where no information processing relationship exists, individuals are limited to invoking privacy to safeguard their personal information. Nevertheless, in contexts where such a relationship exists, they can seek protection through privacy rights, specific personal information protection clauses, or even pursue public interest litigation.^53^ The civil law framework for personal information protection upholds the principle of information self-determination, reflecting individual autonomy through mechanisms like informed consent, information disclosure, and the safeguarding of individual rights.^54^

The *Civil Code* ensures the protection of personal information rights and reflects the attributes of safeguarding societal public interests and managing collective risks, as demonstrated by the civil public interest litigation case *Hangzhou Shangcheng District People’s Procuratorate v. Sun*, which encompassed the illegal trading of personal information.^55^ Nonetheless, disputes over the boundaries of information rights under the Chinese Civil Code persist, limiting its full effectiveness, as evidenced by the civil judgment in China’s first facial recognition case *Guo Bing v. Hangzhou Safari Park*.^56^ Studies analyzing numerous civil cases involving personal information have revealed ambiguities in delineating personal information rights versus privacy rights, with notably low compensation for infringements.^57^ Legal research on facial recognition information has highlighted risks in civil law practice, encompassing the potential failure of informed consent rules, difficulty in establishing damages for civil remedies, and the ineffectiveness of guardian consent rules.^58^ In civil law, NPI can be protected under both personal information rights and privacy rights. NPI may include public information, such as widely acknowledged theories and laws, and private information, such as extensive unexpressed thoughts, feelings, or consciousness. For publicly disclosed NPI, individuals should have the right to delete or modify it. Regarding private information within NPI, individuals can exercise their privacy rights or assert both privacy and personal information rights, as long as the content is confidential and intended to remain undisclosed.

### III.C. Administrative Protection of Personal Information

The administrative law domain provides extensive, targeted, and comprehensive provisions for the protection of personal information. In 2013, China introduced its first departmental regulation specifically focused on this issue, the *Provisions on Protecting the Personal Information of Telecommunications and Internet Users*. This was followed by the enactment of the *Cybersecurity Law* in 2016, the *Data Security Law* in 2021, and the PIPL in 2021.^59^ Among these, the PIPL, officially adopted in August 2021, represents China’s first comprehensive and systematic statute in this field. It also addresses the issue of previous fragmented legislation and establishes a standard framework for personal information protection. The PIPL demonstrates a strong administrative law character, which is primarily reflected in three key aspects: (i) State organs, as significant processors of personal information, are bound by the provisions of the PIPL; (ii) A dedicated chapter within the PIPL outlines specific rules for state organs when processing personal information; and (iii) The PIPL includes various provisions concerning administrative penalties.^60^ The following discussion will focus on the administrative rules established within the PIPL. Unlike other legal frameworks, the PIPL addresses sanctions or remedies following rights infringements and governs the entire lifecycle of personal information from collection to circulation. In contrast to criminal and civil laws, administrative law prioritizes the integrity and systematization of personal information protection, offering the benefits of speed and efficiency.^61^

The PIPL adopts a principle of prohibition with specific exceptions for processing personal information, setting strict standards for the legality, legitimacy, and necessity of such processing.^62^ For instance, in the case of *Wu v. Shanghai X Information Service Co., Ltd*, the court ruled that the e-commerce platform’s provision of user information to third parties constituted an infringement without a legal basis.^63^ This law emphasizes the protection of personal rights and interests, imposing stringent oversight on information processors. In practice, the rules on personal information in administrative law constitute a set of legal orders focused on state-led risk control, actively shaped and safeguarded by public regulation.^64^

Under the PIPL, NPI qualifies as sensitive personal information. The identification of sensitive personal information hinges on recognizing its associated risks, specifically its potential impact on the dignity, safety, and property of an individual. The typical categories of sensitive personal information encompass biometric data, religious beliefs, specific identity, medical records, and financial accounts.^65^ NPI, which is derived directly from human brain activity, may include private information such as health status, religious beliefs, emotional state, and financial data. The disclosure of such information can lead to discrimination and stigmatization, resulting in social isolation, prejudice, and unfair treatment, thereby undermining personal dignity and self-respect. Thus, NPI qualifies as sensitive personal information and is protected under the PIPL. Moreover, much of NPI falls within the main category of sensitive personal information, such as specific identities and biometrics. NPI may also encompass contents like religious beliefs, travel tracks and medical health contents, all explicitly recognized as sensitive personal information by the PIPL. Therefore, NPI is subject to the protections afforded to sensitive personal information under the PIPL.

## IV. UNCERTAINTIES IN THE LEGAL PROTECTION OF NPI

While NPI is potentially protected under the personal information protection laws of China, this legal protection remains subject to certain uncertainties. In the use of BCIs, NPI is vulnerable to risks such as misuse, hacking, data theft, and other privacy breaches, highlighting the urgent need for proactive measures on informed consent, data security, and personal privacy. Given the nascent nature of BCIs, there is presently a lack of a comprehensive regulatory framework for neurotechnology to address the above issues. At this point, the main unresolved concerns in the legal protection of NPI are as follows:

### IV.A. Absence of Specific Regulations for NPI

Based on prior analysis, NPI should be categorized as sensitive personal information under the PIPL. Nonetheless, the law lacks clear classification or specific clauses for NPI. While the PIPL offers protection for sensitive information, it does not specifically mention NPI, asserting that Chinese legislators may have overlooked the profound privacy risks associated with it.^66^ Various legal debates exist regarding how NPI fits within the existing legal framework and how future laws should be implemented to define and protect neuroprivacy, especially considering that neuroscience may drastically alter the legal status quo.^67^ The personal information protection system of China has not yet established tort rules for NPI infringement although academia has recognized the legal gap surrounding NPI.

Moreover, rules on sensitive personal information in the PIPL are largely general, typically requiring detailed low-level regulations for practical implementation. Nonetheless, regulations specific to protecting NPI remain undeveloped. Unlike ordinary information, NPI can offer insight into the mental state, preferences, and potential future behaviors of a person, resulting in numerous privacy concerns.^68^ Regarding relevance to the human mind, NPI is at least no less sensitive than biometric information. For biometric information, like facial recognition data, China’s Supreme People’s Court issued the judicial interpretation^69^ for providing detailed data collection, use, and transfer.^70^ Conversely, NPI, with potentially even greater sensitivity than biometric information, still awaits protective regulations. While it might be classified as sensitive personal information, the absence of detailed standards for tort and criminal liability may severely limit its protection. A similar gap previously emerged with identity card (ID) information, where unified standards for conviction and sentencing were scarcely in place until the issuance of Guiding Case^71^ No. 193.^72^ Considering the distinct privacy risks linked to NPI, implementing appropriate legal measures is increasingly urgent.

### IV.B. Inadequacy of Traditional Informed Consent Rules

Under the PIPL, processors of sensitive personal information must inform individuals about the necessity of data processing and its impact on individual rights and interests, securing explicit consent from individuals.^73^ The static consent approach under the PIPL does not adequately safeguard the rights of BCI users. NPI collected by BCIs is dynamic and subject to continuous updates over time. The informed consent rules only offer necessary information at specific points, overlooking the continuous evolution of NPI. Once initial consent is given, BCI providers may discover NPI’s novel uses and alter their information processing methods, creating unforeseen privacy risks. While the PIPL allows individuals to withdraw their consent at any time, exercising this right is generally difficult due to the information asymmetry between data subjects and information processors. The emerging and rapidly evolving nature of implantable BCI research complicates the identification of risks, a critical component of informed consent.^74^ Studies on closed-loop BCIs have affirmed that many users find it challenging to fully understand the associated risks.^75^

Patients’ misunderstandings and elevated expectations of BCI treatment outcomes may prompt them to provide informed consent without a full understanding of the risks involved.^76^ The authenticity and validity of such informed consent are thus questionable, considering that BCI patients typically suffer from compromised cognitive status and that the nature of data collection and usage in BCIs significantly differs from traditional treatments.^77^ Limited professional knowledge generally hinders ordinary citizens from fully assessing the risk of NPI leakage or detecting rights violations during the use of BCIs. Additionally, the uncertainty surrounding the benefits of BCIs and the nature of potential risks add further challenges in obtaining genuine informed consent.^78^ In summary, the dynamic nature of NPI, the unpredictability of potential risks, and the complexity of BCIs present a series of challenges to the informed consent rules under the PIPL. The existing informed consent rules primarily emphasize static consent and do not adequately protect NPI throughout BCI use. Even timely detection and withdrawal of consent by individuals do little to mitigate the potential harm, particularly concerning human dignity and personal reputation.

### IV.C. Potential Risks in NPI Processing

Both the *Civil Code* and the PIPL prescribe principles of legality, fairness, and necessity in the processing of information. The PIPL also mandates necessary measures to prevent data breaches, alterations, or losses, such as encryption and de-identification.^79^ Nonetheless, these general regulations are inadequate for the protection of NPI. Unlike traditional personal information, NPI has the potential to reveal the inner thoughts and unconscious reactions of an individual. In addition to identifying data subjects, NPI may also be employed to influence personal decisions and individual safety. Meanwhile, advancements in computing technology could affect the identifiability of NPI due to its potential. For instance, even if original NPI data is not recognizable, adjustments or combinations with other data may reveal the user’s identity.^80^ Even after undergoing standard anonymization, the re-identification of individuals through NPI remains a possibility.^81^ Research has confirmed that technical processing of de-identified neuroimaging data can expose personal identities and even decode details such as emotional states.^82^^,^^83^

While the risk of re-identification is a common issue across diverse personal information types, the characteristics of NPI make this issue even more complex and sensitive. NPI is more closely tied to human free will and privacy compared with other personal data. Originating from the neural structure of individuals, NPI can reflect deep psychological processes, emotional states, and even unconscious behaviors. The re-identification of NPI may reveal personal identities and expose deeply private internal states or intentions. Once NPI is re-identified, it may cause irreparable harm to the dignity of an individual. Standard anonymization methods utilized for traditional personal information might not meet the unique requirements of NPI protection. The absence of specific provisions for NPI in the PIPL implies that anonymized NPI could be improperly collected, employed, and shared, potentially resulting in privacy breaches and autonomy violations, along with risks of discrimination based on individual differences.^84^

### IV.D. Deficiencies in Government Data Access Mechanism

The PIPL sets clear boundaries for entities like businesses in processing personal information but provides limited guidance for regulating government agencies that may access such information. Technologies like functional magnetic resonance imaging (fMRI) are expected to play a significant role in detecting neural patterns linked to deception or other criminal behaviors in legal contexts.^85^ ‘Brain fingerprinting’ techniques are already utilized to identify lies in suspects.^86^ Nonetheless, the use of brain investigations in law enforcement raises serious concerns about potential infringements on privacy and cognitive freedom.^87^ The PIPL and related personal information protection laws have long struggled with weak regulatory oversight of government agencies, as seen in limited supervision and enforcement measures. Especially in the use of NPI, insufficient government regulation could result in serious privacy concerns and potentially threaten individual autonomy owing to the strong link between NPI and cognitive processes. These unique characteristics of NPI highlight the need for stronger protections, potentially serving as a catalyst for necessary legal reforms.

Moreover, the utilization of BCIs in government enforcement poses risks of legally gathering and storing large amounts of NPI. Some BCI applications require the collection and analysis of complete raw EEG data during operation.^88^ Such data could potentially be employed for numerous applications, encompassing identity identification,^89^ mental health diagnostics,^90^ and emotion tracking.^91^ Excessive collection of personal information during the use of BCIs could result in privacy breaches, particularly in the absence of relevant regulatory standards.^92^ Within the current Chinese legal framework, insufficient oversight of government agencies may allow for improper use of NPI, resulting in potential infringements on individual rights. Furthermore, BCI technology development currently lacks extensive regulatory precedents, and the extended legislative process may enable certain malpractices and gray areas to persist.

## V. RECOMMENDATIONS FOR STRENGTHENING THE LEGAL PROTECTION OF NPI

The current legislative challenge in protecting NPI emerges both from oversight gaps and the inherent limitations of hard law. Hard law refers to precise rules capable of imposing legally binding obligations on others, with enforcement powers granted to third-party decision-makers.^93^ While hard law offers substantial enforcement benefits, the opacity of emerging technology research and development, coupled with legislative delays, may hinder traditional hard law from effectively safeguarding NPI.^94^ At this juncture, soft law can address these shortcomings by providing flexibility and diversity. Soft law refers to rules of conduct that, while not legally binding, can still exert practical influence.^95^ Compared with hard law, soft law is less costly to formulate, can be updated more quickly, and offers a wider range of regulatory approaches, allowing for greater flexibility in adapting to evolving social contexts.

**Figure 1:**
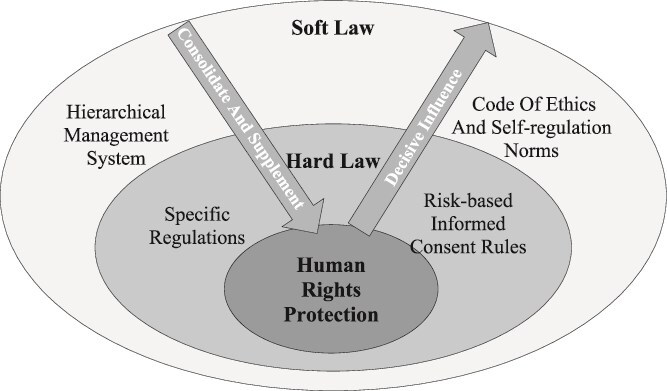
The Concentric–Circle Hard–Soft Law Continuum for NPI Protection

Soft law and hard law are not mutually exclusive but instead work together in achieving social governance objectives.^96^ In the continuum of hard and soft law, state-enacted hard law holds the central position within the system structure, forming a ‘center-periphery’ model of mixed hard–soft law governance.^97^ For the protection of NPI, the current study proposes a human rights-centered approach, constructing a concentric-circle legal continuum of hard and soft law (Fig. 1). This approach promotes a multi-center governance approach, enabling communication and collaboration among government, society, the market, and other stakeholders.

### V.A. Enhancing Specific Regulations

Currently, China’s legislation does not offer a clear definition and classification for NPI, leading to interpretative ambiguities and practical loopholes. The current legal framework does not clearly define the scope of NPI, thereby creating a legal blind spot in information protection. Defining NPI is technically difficult, but clarifying its legal scope is feasible by listing its main categories and including a catch-all clause.^98^ In principle, the scope of NPI should encompass, but not be limited to the following: (i) internal mental information, such as thoughts, beliefs, memories, values, emotions, mental states, and other private data related to brain activities; (ii) health conditions and medical information, involving information on diseases, diagnoses, treatments, and medications; and (iii) raw neurological data collected from various sources such as EEG recordings, fMRI data, and genetic data related to neurological conditions. Specifically, considering that anonymized NPI can still be re-identified, it should also be covered under legal protection. Legally clarifying the scope of NPI helps in enhancing the comprehensiveness and precision of the information protection framework, raising public awareness of NPI protection.

The current tort and criminal regulations regarding NPI infringements are vague, potentially leading to challenges in judicial processes. Given the high sensitivity of NPI, its infringement assessment could adopt a ‘default-high risk’ principle: unauthorized access to NPI and any actions based on it, such as unauthorized collection and transmission, should be presumed to constitute an infringement. Additionally, a tiered liability system for NPI infringements is proposed, distinguishing between minor and severe infringements based on the degree of infringement and the harm caused. Minor NPI infringements could incur civil liability and require compensation, whereas severe NPI infringements might be considered criminal offenses, with penalties scaled according to the damage caused. Judicial interpretations or guiding cases could establish specific standards for the conviction and sentencing of such offenses, taking into account the volume of information and the amount of illegal profits involved. Additionally, supplementary ethical guidelines, such as the Measures for the Review of Science and Technology Ethics (Trial) issued in 2023, could further safeguard NPI.

### V.B. Establishing a Hierarchical Management System

The establishment of a hierarchical and classified system for NPI is an effective approach to mitigate the privacy risks of BCIs. Based on content and potential usage, NPI can be classified into the following three types: highly identifiable NPI, sensitive NPI, and general NPI.

Highly identifiable NPI comprises neural data that is easily recognizable and has the potential to disclose personal identity, such as brainwave patterns and cerebral cortex signals. This type of data, with biometric capabilities, requires the highest level of protection and stringent management measures. Effective data management should include the implementation of highly secure storage systems, strong encryption, and multilayered access controls to prevent unauthorized access and data breaches. To safeguard NPI from cyberattacks, information processors should implement rigorous cybersecurity measures and conduct regular audits to ensure data integrity and security.

Sensitive NPI encompasses neural data that, while less recognizable, holds significant personal information, such as thoughts, emotions, and memories. Although this type of data may not perform biometric functions, it contains substantial information, and its exposure could undermine personal dignity and autonomy. Thus, sensitive NPI necessitates robust oversight, with data being promptly deleted after use. It is recommended to establish short-term storage rules for sensitive NPI to ensure timely data deletion after use. Information processors should utilize effective technical measures to limit data retention duration and specify usage purposes, thereby reducing potential privacy risks. Additionally, the information protection department should enforce strict supervision over the collection and use of sensitive NPI to ensure that information processing remains lawful and compliant.

General NPI refers to routine neural data that do not fall into the above two categories, including meaningless daily logs and publicly available data. This type of data, being less identifiable and comprises fewer private details, generally must be integrated with other data to yield useful information. Consequently, NPI should be subject to essential regulatory measures and safeguarded from unauthorized disclosure through appropriate data security protocols. General NPI management can be streamlined, but information processors must undertake fundamental data security measures to prevent privacy risks from data leaks.

Differentiated protection measures for various types of NPI can elevate the precision of NPI safeguards while supporting BCI technology development. This hierarchical classification system provides a promising approach to better balancing information accessibility and privacy protection.

### V.C. Strengthening the Risk-Based Informed Consent Rules

The current informed consent rules for personal information focus on obtaining prior individual consent, which struggles to address emergencies during BCI use.

From a static perspective, a hierarchical consent model for NPI should be established, focused on risk prevention:

For highly identifiable NPI and sensitive NPI, such as thoughts and consciousness, general collection and use should be prohibited. Consent should only be obtained after government approval in special circumstances. These special circumstances encompass scenarios involving national security, significant public interest, and emergency medical assistance. In such cases, the use of highly identifiable and sensitive NPI may be permitted following a rigorous vetting process, but only to the extent necessary to fulfill the specified purpose.

For general NPI, a robust protection model with special consent requirements should be implemented, thereby establishing a comprehensive consent system encompassing the entire information processing lifecycle, with separate informed consent required for each instance of data collection, use, and sharing. This requirement also extends to special circumstances. Considering that NPI may undergo multiple stages of processing, sharing, and transmission during use, its privacy risks and purposes may change over time, creating new challenges in distinct contexts. Obtaining informed consent in stages and on a case-by-case basis helps in ensuring that individuals have full knowledge and control over the processing of their NPI. This measure also serves as a crucial tool to prevent the misuse of information in a complex digital environment.

For NPI that has been actively disclosed, a more flexible protection model with an opt-out option should be applied, allowing individuals to withdraw their disclosed information at any time. Information processors should provide straightforward opt-out mechanisms, such as a one-click opt-out option within a platform or application. Similar data governance practices can be found in the consent revocation mechanisms under the GDPR framework.^99^ Tools like BCI Anonymizer should be employed to protect NPI whenever possible.^100^ The cost of implementing these informed consent protocols should be borne by BCI providers.

From a dynamic perspective, a comprehensive system-wide response mechanism for risk warnings should be established at every stage of the design, manufacture, adoption, and use of BCI equipment, accompanied by risk control measures.^101^ When significant risks are identified, information processors must promptly notify individuals and obtain updated informed consent. Stakeholders should ensure that relevant policies are both well informed and straightforward.^102^

### V.D. Developing Codes of Ethics and Self-Regulation Norms

The rapid development of BCIs necessitates the establishment of robust ethical standards and self-regulatory norms within the neurotechnology industry, particularly regarding information protection. BCI device manufacturers, technology developers, and clinical trial organizations must establish ethics committees. These committees should regularly oversee adherence to relevant standards, policies, and rules of conduct to ensure comprehensive respect for the rights of users. Regular reviews of official policies, laws, regulations, and industry standards should be conducted, with timely updates to ethical norms as necessary.^103^ The ethics committee may also appoint ethics officers to ensure that all aspects of the organization’s procedures align with established ethical codes.^104^

Governments can incentivize companies to establish ethics committees by offering tax breaks or other financial incentives.^105^ External social reputation can motivate companies to comply with ethical regulations. Establishing an ethics committee and upholding rigorous ethical standards can enhance consumer and investor trust, strengthening a company’s brand value.^106^ By adopting soft law, companies can enhance their public image and market reputation, thereby attracting more stakeholders and investors.^107^ The democratic nature of soft law governance enables emerging technology companies to implement effective internal governance.^108^ This fosters healthy competition among business organizations, as they may attract consumers by adopting soft laws that are more favorable to the public.

Furthermore, the establishment of a self-regulatory system in the neurotechnology industry will be a critical measure to enhance the protection of NPI. This requires broad cooperation among stakeholders, encompassing consensus on codes of conduct, standardization of technical benchmarks, and the construction of third-party audit and certification systems.^109^ For instance, the BCI industry association can collaborate with third-party certification entities to uniformly review the information handling practices of various BCI companies and classify them accordingly. Non-governmental organizations, think tanks, and journalists could oversee and publicly report on compliance with certain soft law programs and monitor any misconduct by public authorities.^110^ This strategy would support governmental oversight and leverage public opinion to encourage standardized development in the neurotechnology field.

## VI. CONCLUSION

NPI qualifies as sensitive personal information and is hence protected under the personal information legislation of China. Nevertheless, existing Chinese laws lack detailed provisions to offer effective protection for NPI, particularly regarding anti-infringement measures and standards for conviction and sentencing. Current informed consent rules, information processing standards, and regulatory framework fall short of addressing the privacy threats inherent in neurotechnology. To address these gaps, a concentric-circle continuum of hard and soft law can be instrumental in fostering the standardized development of neurotechnology. In hard law, further refinement of NPI regulations and enhancement of risk-based informed consent rules are essential. In soft law, establishing a hierarchical classification management system for NPI, enriching the ethical norms in information processing, and encouraging industry self-regulation in the neurotechnology industry are essential.

Importantly, the goal of protecting NPI is not to hinder the progress of neurotechnology but to foster a regulated, collaborative environment among scientists, technologists, policymakers, industry leaders, and other stakeholders. Such protections could build public trust in neurotechnology, facilitating accelerated neuroscience research while ensuring the rights and interests of individuals. Neurotechnology represents the cutting-edge integration of neuroscience, artificial intelligence, and digital technology, poised to introduce new impacts and challenges to society. While NPI research remains in its early stages, its significance is undeniable, with the potential for increasing global focus on the topic in the future.

## Footnotes

1 Joseph N. Mak & Jonathan R. Wolpaw, *Clinical Applications of Brain–Computer Interfaces: Current State and Future Prospects*, 2 Ieee  Rev. Biomed. Eng. 187, 187 (2009).

2 See Nature News, *Second brain implant by Elon Musk’s Neuralink: will it fare better than the first*, https://www.nature.com/articles/d41586-024-02368-8 (accessed Oct. 24, 2024).

3 Suzanne Oosterwijk et al., *States of mind: Emotions, body feelings, and thoughts share distributed neural networks*, 62 Neuroimage 2110, 2110 (2012).

4 Vlek Rutger J. et al., *Ethical Issues in Brain–Computer Interface Research, Development, and Dissemination*, 36 J. Neurol. Phys. Ther. 94, 98 (2012).

5 Rafael Yuste et al., *Four Ethical Priorities for Neurotechnologies and AI*, 551 Nature 159, 161 (2017).

6 Marcello Ienca & Pim Haselager. *Hacking the brain: brain–computer interfacing technology and the ethics of neurosecurity*, 18 Ethics  Inf. Technol. 117, 117 (2016).

7 Ofir Landau et al., *Mind Your Privacy: Privacy Leakage Through BCI Applications Using Machine Learning Methods*, 198 Knowl. Based. Syst. 105932, 105932 (2020).

8 See Bonaci Tamara et al., *App stores for the brain: Privacy and security in Brain–Computer Interfaces*, 2014 Ieee  Ethics. 1, 1–7 (2014).

9 Marcello Ienca & Roberto Andorno, *Towards New Human Rights in the Age of Neuroscience and Neurotechnology*, 13 Life  Sci. Soc. Policy. 1, 12 (2017).

10 Marcello Ienca & Gianclaudio Malgieri, *Mental Data Protection and the GDPR*, 9 J. Law  Biosci. lsac006, 1 (2022).

11 Stephen Rainey et al., *Is the European Data Protection Regulation Sufficient to Deal with Emerging Data Concerns Relating to Neurotechnology?*, 7 J. Law  Biosci. lsaa051, 18 (2020).

12 See Nirmala Devi Kathamuthu et al., *Deep Q-Learning-Based Neural Network with Privacy Preservation Method for Secure Data Transmission in Internet of Things (IoT) Healthcare Application*, 11 Electronics 157, 157 (2022).

13 See Jana I. Münßinger et al., *Brain Painting: First Evaluation of a New Brain–Computer Interface Application with ALS-Patients and Healthy Volunteers*, 4 Front. Neurosci. 182, 182 (2010).

14 See Stephanie Naufel & Eran Klein, *Brain–Computer Interface (BCI) Researcher Perspectives on Neural Data Ownership and Privacy*, 17 J. Neural. Eng. 016039, 016039 (2020).

15 See Paul McCullagh et al., *Ethical Challenges Associated with the Development and Deployment of Brain Computer Interface Technology*, 7 Neuroethics. 109, 109–22 (2014).

16 Li Tian, Jialin Zheng & Lize Xiong, *Current Status and Prospects in Brain Research Projects*, 12 Chin. J. Anesthesiol. 8, 8 (2021).

17 See Xiaoling Wei et al., *Nanofabricated Ultraflexible Electrode Arrays for High-density Intracortical Recording*, 5 Adv. Sci. 1700625, 1700625 (2018).

18 See Xiao Yang et al., *Bioinspired Neuron-Like Electronics*, 18 Nat. Mater. 510, 510–17 (2019).

19 See Yonghao Chen et al., *Implementing a Calibration-Free SSVEP-Based BCI System with 160 Targets*, 18 J. Neural. Eng. 046094, 046094 (2021).

20 See Weiwei Yang et al., *The Effect of Brain–Computer Interface Training on Rehabilitation of Upper Limb Dysfunction After Stroke: A Meta-Analysis of Randomized Controlled Trials*, 15 Front. Neurosci. 766879, 766879 (2022).

21 See Hao Fang & Yuxiao Yang, *Predictive Neuromodulation of Cingulo-Frontal Neural Dynamics in Major Depressive Disorder Using a Brain–Computer Interface System: A Simulation Study*, 17 Front. Comput. Neurosc. 1119685, 111968 (2023).

22 See Hai Wang et al., *Automatic Sleep Staging Method of EEG Signal Based on Transfer Learning and Fusion Network*, 488 Neurocomputing 183, 183–93 (2022).

23 See Artificial Intelligence Industry Alliance, *2021 White Paper on the Application of Brain–Computer Interface Technology in Healthcare*, https://pdf.dfcfw.com/pdf/H3_AP202107221505332039_1.pdf?1626969678000.pdf (accessed Apr. 12, 2024).

24 See Foresight Industry Research Institute, *Summary, Interpretation and Development Goals of National and Provincial Brain–Computer Interface Industry Policies in 2023*, https://bg.qianzhan.com/trends/detail/506/221129-ba7c0c14.html (accessed Apr. 14, 2024).

25 See Cyzone Research Center, *2023 Research Report on China Brain–Computer Interface Industry*, http://www.doc88.com/p-69816378202186.html (accessed Apr. 14, 2024).

26 See China Commercial Industry Research Institute, *China Brain–Computer Interface Industry Market Prospects Forecast Research Report 2024*, https://www.163.com/dy/article/IRSD3LEO051481OF.html (accessed Apr. 15, 2024).

27 Cyzone Research Center, *supra* note 25.

28 China Commercial Industry Research Institute, *supra* note 26.

29 See Yuanwen Jiang et al., *Topological Supramolecular Network Enabled High-Conductivity, Stretchable Organic Bioelectronics*, 375 Science 1411, 1411–17 (2022).

30 See China Electronics Standardization Institute, *Brain–Computer Interface Standardization White Paper (2021 Edition)*, https://www.xdyanbao.com/doc/z5y69zmq9u?bd_vid=12047898804583948172 (accessed Apr. 15, 2024).

31 See China Science Daily, *World’s first non-human primate interventional brain–computer interface successfully tested*, https://news.sciencenet.cn/htmlnews/2023/5/500018.shtm (accessed Apr. 16, 2024).

32 See Yu Pei et al., *A Tensor-Based Frequency Features Combination Method for BCIs*, 30 Ieee  Trans. Neural  Syst. Rehabilitation  Eng. 465, 465–75 (2022).

33 See Jing Jin et al., *A Novel Classification Framework Using the Graph Representations of Electroencephalogram for Motor Imagery Based Brain–Computer Interface*, 30 Ieee  Trans. Neural  Syst. Rehabilitation  Eng. 20, 20–29 (2022).

34 See Xun Wu et al., *Investigating EEG-Based Functional Connectivity Patterns for Multimodal Emotion Recognition*, 19 J. Neural. Eng. 016012, 016012 (2022).

35 See Yifan Miao et al., *MLDA: Multi-Loss Domain Adaptor for Cross-Session and Cross-Emotion EEG-Based Individual Identification*, 27 Ieee j. Biomed. Health 5767, 5767–78 (2023).

36 See Shugeng Chen et al., *Exploration of the Non-invasive Brain–Computer Interface and Neurorehabilitation*, 18 Front. Neurosci. 1377665, 1377665 (2024).

37 See Linfeng Cao et al., *A Brain-Actuated Robotic Arm System Using Non-invasive Hybrid Brain–computer Interface and Shared Control Strategy*, 18 J. Neural. Eng. 046045, 046045 (2021).

38 See Zuoting Song et al., *Evaluation and Diagnosis of Brain Diseases Based on Non-invasive BCI*, 9 Ieee  Int. Win. Conf. Bci. Proc. 1–6 (2021).

39 Artificial Intelligence Industry Alliance, *supra* note 23.

40 See Kejun Xie et al., *Portable Wireless Electrocorticography System with a Flexible Microelectrodes Array for Epilepsy Treatment*, 7 Sci. Rep. 7808, 7808 (2017).

41 See China Business Industry Research Institute, *China BCI Industry Development Trend and Investment Risk Research Report 2022–2027*, https://www.163.com/dy/article/IFO8CDD1051481OF.html (accessed Apr. 18, 2024).

42 China Commercial Industry Research Institute, *supra* note 26.

43 Janis J Daly & Jonathan R Wolpaw, *Brain–Computer Interfaces in Neurological Rehabilitation*, 7 Lancet  Neurol. 1032, 1032 (2008).

44 Niels Birbaumer et al., *Neurofeedback and Brain–Computer Interface: Clinical Applications*, 86 Int. Rev. Neurobiol. 107, 107 (2009).

45 See Xiang Chen et al., *Design and Implementation of Human–Computer Interaction Systems Based on Transfer Support Vector Machine and EEG Signal for Depression Patients’ Emotion Recognition*, 11 J. Med. Imag. Health  In. 948, 948–54 (2021).

46 See Jian Zhang, Ke Li & Yang Feng, *Criminal Sanctions on Identity Theft in Shanghai: An Empirical Case Law Analysis*, 71 Int. J. Law  Crime  Justice 100562, 100,562 (2022).

47 Dezheng Wang, *From Classification to Hierarchy: Criminal Law Protection of Citizens’ Personal Information in China*, 5 Hunan  Soc. Sci. 122,128 (2023).

48 Dongyan Lao, *Models of Criminal Law Protection of Personal Data*, 12 Soc. Sci. Digest 73, 74 (2020).

49 Yong Pi, *On the Duality of Legal Interests of Personal Information in Criminal Law*, 4 Soc. Sci. Res. 74, 74 (2023).

50 See Yang Feng, Ke Li & Jian Zhang, *An Empirical Study of Criminal Sanctions on the Crime of Infringing Personal Information in China*, 1 J. Shandong  Univ. (Philos. Soc. Sci.) 92, 95–98 (2023).

51 Jian Zhang, Ke Li & Yang Feng, *supra* note 46, at 100562.

52 Dezheng Wang, *supra* note 47, at 129.

53 Xiaodong Ding, *The Jurisprudence of the Relationship Between the Protection of the Right to Privacy and Personal Information—With a Discussion on the Application of Civil Code of P.R.C and the Personal Information Protection Law of P.R.C.*, 40 Stud. Law  Bus. 61, 61 (2023).

54 Peiru Cai, *The Distinction of Personal Information Protection Principles: Protection by Process and Protection as a Result*, 5 Admin. L. Rev. 91, 92 (2021).

55 See Hangzhou Internet Court, *Top Ten Typical Cases of Personal Information Protection,*  https://www.secrss.com/articles/46037 (accessed Apr. 20, 2024).

56 Chuanlin Lin & Zongyu Song, *Civil Law Positioning and Path Selection for Personal Information Protection*, 4 Gansu  Soc. Sci. 206, 206 (2021).

57 See Yun Wo & Pengfei Qiao, *Judicial Investigation and System Improvement of Personal Information Protection under the Background of ‘Civil Code’*, 5 Credit  Ref. 31, 35–36 (2024).

58 See Yuying Wang, *Exploration for Protection of Personal Information in Facial Recognition*, 2 J. Law  Appl. 15, 18–21 (2023).

59 Lixin Zhu & Chuyun Qi, *Ten-Year Review of the Legal Regime for the Protection of Personal Information*, 2 China  Inf. Secur. 71, 72 (2023).

60 Aimin Qi, *The Administrative Law Attributes of Personal Information Protection Law*, 2 J. Party  Sch. Changchun  Comm. Cpc. 73, 73 (2008).

61 Chunye Wang & Bo Fei, *Administrative Regulations Governing the Collection and the Use of Personal Information in the Context of Big Data*, 23 J. Party  Sch. Tianjin  Comm. Cpc. 72, 72 (2021).

62 Xu Yang, *Commentary on Art. 13(1) of the Chinese Personal Information Protection Law (Legal Basis of Personal Information Processing)*, 6 China j. Appl. Jurisprud. 202, 203 (2023).

63 Hangzhou Internet Court, *supra* note 55.

64 Xixin Wang, *The Three-level Frame and Protection Mechanism of Personal Information Rights and Interests*, 43 Mod. Law  Sci. 105, 117 (2021).

65 See Standing Committee of the National People’s Congress of P. R. C., *Chinese Personal Information Protection Law,* (last reviewed Aug. 20, 2021), http://www.npc.gov.cn/npc/c30834/202108/1d133c79437b4720a0c3b3ce29f5d5e2.shtml (accessed Apr. 16, 2024).

66 See Marcello Ienca & Roberto Andorno, *supra* note 9, at 5.

67 Dominique J. Church, *Neuroscience in the Courtroom: An International Concern*, 53 Wm. & Mary L. Rev. 1825, 1830 (2012).

68 See Jwa Anita S. & Russell A. Poldrack, *Addressing privacy risk in neuroscience data: from data protection to harm prevention*, 9 J. Law  Biosci. lsac025, lsac025 (2022).

69 Judicial interpretation is one type of documents issued by the Supreme People’s Court, which aims to either comprehensively interpret provisions of national laws or answer specific questions deriving from the judicial practice of local courts. It is regarded as a source of law for the court to adjudicate cases. For a detailed analysis of the status and effect of judicial interpretation, see Eric C. Ip, *Supreme People’s Court and the Political Economy of Judicial Empowerment in Contemporary China*, 24 The  Colum. J. Asian L. 239, 239–405 (2010).

70 See Supreme People’s Court of China, *Provisions of the Supreme People’s Court on Several Issues Concerning the Application of Law in the Trial of Civil Cases Relating to Processing of Personal Information by Using the Facial Recognition Technology,* (last reviewed June 27, 2021), https://www.pkulaw.com/en_law/452f945c46c4739fbdfb.html (accessed Nov. 5, 2024).

71 A ‘guiding case’ refers to a specific type of legal precedent that courts utilize to interpret laws and provide direction to lower courts. This concept is particularly important in China, where the Supreme People’s Court (SPC) issues guiding cases to enhance the consistency of legal applications and improve judicial quality. See Jia Mark, *Chinese common law? Guiding cases and judicial reform,* 129 Harv. Law  Rev. 2213, 2213 (2016).

72 See Supreme People’s Court of China, *Guiding Case No. 193: Case of Infringing on Personal Information of Citizens by Wen Wei et al.,* (last reviewed Dec. 26, 2022), https://www.pkulaw.com/en_law/452f945c46c4739fbdfb.html (accessed Nov. 5, 2024).

73 See Standing Committee of the National People’s Congress of P. R. C., *supra* note 65.

74 Eran Klein, *Informed Consent in Implantable BCI Research: Identifying Risks and Exploring Meaning*, 22 Sci. Eng. Ethics 1299, 1299 (2016).

75 Eran Klein et al., *Brain–Computer Interface-Based Control of Closed-Loop Brain Stimulation: Attitudes and Ethical Considerations*, 3 Brain–Computer  Interfaces 140, 145 (2016).

76 Leigh Hochberg & Thomas Cochrane, *Implanted Neural Interfaces: Ethics in Treatment and Research*, in Neuroethics  in  Practice. Medicine, Mind, and  Society 241 (A. Chatterjee & M. J. Farah ed., 2013).

77 Laman Yusifova, *Ethical and Legal Aspects of Using Brain–Computer Interface in Medicine: Protection of Patient’s Neuro Privacy*, Univ. Di  Bologna  Ph.d. Thesis, 65 (2020).

78 Erica Palmerini, *A Legal Perspective on Body Implants for Therapy and Enhancement, International Review of Law*, 29 Comput. Technol. 226, 234 (2015).

79 See Standing Committee of the National People’s Congress of P. R. C., *supra* note 65.

80 Stephen Rainey et al., *supra* note 11, at 13.

81 Marcello Ienca et al., *Towards a Governance Framework for Brain Data*, 15 Neuroethics 20, 25 (2022).

82 Christopher G. Schwarz et al., *Identification of Anonymous Mri Research Participants with Face-Recognition Software*, 381 N. Engl. J. Med 1684, 1684 (2019).

83 Ahmet Omurtag, Haleh Aghajani & Hasan Onur Keles, *Decoding Human Mental States by Whole-Head EEG + fNIRS During Category Fluency Task Performance*, 14 J. Neural. Eng. 066003, 066003 (2017).

84
Frischmann B. M. & Selinger E, The  Digital  Identity  Crisis and the  Dawn of  Data  Dignity, 121 (2021).

85 Giorgio Ganis et al., *Neural Correlates of Different Types of Deception: An fMRI Investigation*, 13 Cereb. Cortex 830, 830 (2003).

86 Nita A Farahany, *Incriminating Thoughts*, 64 Stan. L. Rev. 351, 382 (2012).

87 Arian Petoft & Mahmoud Abbasi, *Current Limits of Neurolaw: A Brief Overview*, 161 Méd. Droit 29, 33 (2020).

88 Brandon King, Gemma Read & Paul Matthew Salmon, *The Risks Associated with the Use of Brain–Computer Interfaces: A Systematic Review*, 40 Int. j. Hum-Comput. Int. 131, 140 (2024).

89 Marios Poulos, Maria Rangoussi & Nikolaos Alexandris, *Neural Network Based Person Identification Using EEG Features*, 1999 Ieee  Int. Conf. Acoust. Speech, Signal  Process. Proc. 1117 (1999).

90 Shu Lih Oh et al., *Deep Convolutional Neural Network Model for Automated Diagnosis of Schizophrenia Using EEG Signals*, 9 Appl. Sci. 2870, 2870 (2019).

91 Kranti Kamble & Joydeep Sengupta, *A Comprehensive Survey on Emotion Recognition Based on Electroencephalograph (EEG) Signals*, 82 Multimed. Tools. Appl. 27269, 27269 (2023).

92 Hassan Takabi, Anuj Bhalotiya & Manar Alohaly, *Brain Computer Interface (BCI) Applications: Privacy Threats and Countermeasures*, 2 Int. Conf. Cic. Proc. 102 (2016).

93 Kenneth W. Abbott & Duncan Snidal, *Hard and Soft Law in International Governance*, 54 Int. Organ. 421, 421 (2000).

94 Matthew U. Scherer, *Regulating Artificial Intelligence Systems: Risks, Challenges, Competencies, and Strategies*, 29 Harv. jl & Tech. 353, 358 (2015).

95 See Abbott Kenneth W. & Duncan Snidal, *Hard and soft law in international governance*, 54 Int. Organ. 421, 421–56 (2000).

96 Cynthia Crawford Lichtenstein, *Hard Law V. Soft Law: Unnecessary Dichotomy*, 35 Int’l L 1433, 1434 (2001).

97
Michael  Byers, The  Role of  Law in  International  Politics, 337 (2000).

98 A catch-all clause is a provision in a contract or legal document that is designed to cover a broad range of unspecified events or circumstances. This clause typically serves as a safety net, ensuring that if a situation emerges that is not explicitly addressed in the agreement, the clause can be invoked to provide a legal remedy or define the rights and obligations of the parties involved. See Kearney J. & Mankin A, *Catch-All Clauses: Their Role in Contractual Agreements*, 65 Bus. Law. 1251, 1251–74 (2010).

99 See Politou E., Alepis E. & Patsakis C., *Forgetting personal data and revoking consent under the GDPR: Challenges and proposed solutions*. 4 J. Cybersecurity tyy001, tyy001 (2018).

100 BCI Anonymizer technology seeks to mitigate risks by anonymizing brain data to protect the personal data of users. Specifically, this technology prevents unauthorized inference of sensitive information, such as mental states or PINs, by anonymizing or obfuscating identifiable data in real time. See Poonam Chaudhary & Rashmi Agrawal, *Emerging Threats to Security and Privacy in Brain Computer Interface*, 3 Int. J. Adv. Res. 340, 343 (2018).

101 Brandon J. King, Gemma JM Read & Paul M. Salmon, *Identifying Risk Controls for Future Advanced Brain–Computer Interfaces: A Prospective Risk Assessment Approach Using Work Domain Analysis*, 111 Appl. Ergon. 104,028, 104,028 (2023).

102 Eran Klein, *supra* note 75, at 1314.

103 Gary Marchant, Lucille Tournas & Carlos Ignacio Gutierrez, *Governing Emerging Technologies Through Soft Law: Lessons for Artificial Intelligence—an Introduction*, 61 Jurimetrics 1, 18 (2020).

104 Henry Adobor, *Exploring the Role Performance of Corporate Ethics Officers*, 69 J. Bus. Ethics 57, 57 (2006).

105 See Ruhnka J. C. & Boerstler H, *Governmental incentives for corporate self-regulation*. 17 J. Bus. Ethics 309, 309–326 (1998).

106 See Stevens J. M., Steensma H. K. & Harrison D. A., *Symbolic or substantive document? The influence of ethics codes on financial executives’ decisions*. 26 Strategic  Mgmt. J. 181, 181–95 (2005).

107 See Williams C. A., *Civil society initiatives and soft law in the oil and gas industry*. 36 Nyu J. Int’l L. & Pol. 457, 457–509 (2003).

108 Carlos Ignacio Gutierrez, Gary Marchant & Lucille Tournas, *Lessons for Artificial Intelligence from Historical Uses of Soft Law Governance*, 61 Jurimetrics 133, 137 (2020).

109 See Gary E. Marchant & Brad Allenby, *Soft Law: New Tools for Governing Emerging Technologies*, 73 Bull. At. Sci. 108, 112–13 (2017).

110 Gary Marchant, *supra* note 103, at 12.

